# Systematic review of how Play Streets impact opportunities for active play, physical activity, neighborhoods, and communities

**DOI:** 10.1186/s12889-019-6609-4

**Published:** 2019-03-22

**Authors:** M. Renée Umstattd Meyer, Christina N. Bridges, Thomas L. Schmid, Amelie A. Hecht, Keshia M. Pollack Porter

**Affiliations:** 1 Department of Public Health, Baylor University, College of Health and Human Sciences, Waco, TX USA; 2 Department of Health, Human Performance, & Recreation, Baylor University, College of Health and Human Sciences, Waco, TX USA; 3 Sr. Advisor Physical Activity and Health Branch, Centers for Disease Control and Prevention, Atlanta, GA USA; 4 Department of Health Policy and Management, Johns Hopkins Bloomberg School of Public Health, Baltimore, MD USA

**Keywords:** Temporary play space, Non-school physical activity, Safe play places, Children, Adolescents, Play

## Abstract

**Background:**

Active play and physical activity are important for preventing childhood obesity, building healthy bones and muscles, reducing anxiety and stress, and increasing self-esteem. Unfortunately, safe and accessible play places are often lacking in under-resourced communities. Play Streets (temporary closure of streets) are an understudied intervention that provide safe places for children, adolescents, and their families to actively play. This systematic review examines how Play Streets impact opportunities for children and adolescents to engage in safe active play and physical activity, and for communities and neighborhoods. Methods for evaluating Play Streets were also examined.

**Methods:**

A systematic literature review was conducted in Academic Search Complete, CINHAL, PsycINFO, PubMED, Web of Science, and Google Scholar. Peer-reviewed intervention studies published worldwide were included if they were published in English, through December 2017 and documented free-to-access Play Streets or other temporary spaces that incorporated a designated area for children and/or adolescents to engage in active play. Systematic data extraction documented sample, implementation, and measurement characteristics and outcomes.

**Results:**

Of 180 reviewed abstracts, 6 studies met inclusion criteria. Studies were conducted in five different countries (*n* = 2 in U.S.), using mostly cross-sectional study designs (*n* = 4). Physical activity outcomes were measured in half of the studies; one used observational and self-report measures, and two used device-based and self-report measures. In general, Play Streets provided safe places for child play, increased sense of community, and when measured, data suggest increased physical activity overall and during Play Streets.

**Conclusions:**

Play Streets can create safe places for children to actively play, with promise of increasing physical activity and strengthening community. Given the popularity of Play Streets and the potential impact for active play, physical activity, and community level benefits, more rigorous evaluations and systematic reporting of Play Streets’ evaluations are needed.

## Background

Physical activity is an important determinant of obesity risk, which is a major public health problem in school-aged children in the United States (U.S.) and globally [1, 2]. In fact, physical inactivity is the fourth leading risk factor for mortality globally [3]. Regular physical activity in childhood and adolescence also helps build healthy bones and muscles, reduces anxiety and stress, increases self-esteem, and may improve blood pressure and cholesterol levels [4]. Yet, nearly 4 out of 5 U.S. children and adolescents do not meet national physical activity guidelines, with the majority of children and adolescents residing in other countries not meeting World Health Organization (WHO) physical activity guidelines (England: 22% of children (5–15 years of age), Australia: 19% of children (5–17 years of age), Belgium: 7% of children (6–9 years of age) and 2% of adolescents (10–17 years of age), Chile: 25% of children (6–9 years of age) on weekdays and 14% on weekend days) [5–10]. Guidelines for physical activity from both the U.S. and WHO, state that children and adolescents should participate in 60 min or more of physical activity per day. Most of the 60 min should be moderate to vigorous aerobic activity. In addition, vigorous aerobic, muscle-strengthening, and bone-strengthening physical activity should be incorporated as part of the required 60 min or more at least 3 days a week [3, 4].

Disparities in physical activity levels and access to opportunities for activity exist [4], making it challenging for some children to meet recommended physical activity levels. Features of the built environment, including sidewalks, parks, connectivity, and traffic patterns have been associated with physical activity among children [11]. In many underserved and under-resourced communities, built environment characteristics positively associated with physical activity (e.g., sidewalks, parks, connectivity, traffic patterns) are often lacking, and spaces for physical activity are more frequently perceived by parents as unsafe for children due to crime and violence [12].

Play Streets is an intervention that can address these inequities and increase access to safe places for physical activity and active play globally. Play Streets are the temporary closure of streets, that for a specified time period (around 3–5 h) create a safe, publicly accessible space for children, adolescents, and/or their families to engage in active play (closures can be recurring or episodic) [13–17]. Most Play Streets are supervised in some capacity, include multiple activity areas using loose or temporary equipment (e.g., hula hoops, inflatable/bounce house, balls/sports, etc.…), and usually occur during summer months. Although Play Streets have gained in popularity in recent years [18], use of these approaches date back to as early as the 1920s in the U.S. and 1930s in the United Kingdom (U.K.) [19–21]. The idea of creating temporary play space in streets or other publicly available spaces (e.g., parking lots) is similar to “pop-up” parks/play areas, and also could be seen as part of larger community events, such as in the Open Streets and Ciclovías. Pop-up parks occur when a segment of a street or a parking lot is closed off and used to create a temporary public space [22]. Unlike most Play Streets, pop-up parks typically include temporary play structures and resemble a park, rather than an open play space with some activities [22]. Ciclovías and Open Streets initiatives are broad all-community events lasting a few hours to a full day, that similar to Play Streets, close a section of, or entire, street(s) to vehicular/motorized traffic. Open Streets and Ciclovía initiatives usually promote community connectivity, walking, jogging, and cycling, while also providing opportunities for residents to engage in and be exposed to other less common physical activity opportunities through “activity hubs” with organized activities (e.g., yoga, dance classes, sports demonstrations, etc.…) [23, 24]. Since little detail is usually reported describing activity hubs, it is possible that some Ciclovía and Open Streets initiatives incorporate a Play Streets “style” component as part of an activity hub to allow for general active play for children, adolescents, and/or their families [23, 24]. In the U.S. and U.K., Play Streets are the most common of these initiatives focused on increasing opportunities for children’s active play, with 660 streets in the U.K. hosting regular Play Streets as of June 2018 and cities in the U.S. such as Chicago hosting over 650 summer Play Streets since 2012, Seattle hosting over 350 since 2013, and San Francisco hosting Play Streets in 2013, 2017, and 2018 [13, 14, 25–27]. In other countries Ciclovía and Open Streets events are more common, with the potential of incorporating Play Streets “style” components as part of activity hubs, such as in Bogotá, Colombia where Ciclovías have been a weekly occurrence since 1974 [28].

Play Streets address health inequities by providing places for safe active play for children and adolescents in neighborhoods without access to safe and/or well-maintained parks and playgrounds [13, 14]. Play Streets also have the potential to help raise awareness and build a culture around the need for safe built environments and traffic safety interventions in communities [13–16]. In addition, Play Streets initiatives have enhanced neighborhoods through partnership building and increasing social cohesion of residents within the community [13–16].

While the present need for Play Streets is apparent in many communities either lacking parks or other built/natural physical activity spaces or facing safety concerns, Play Streets have been implemented in communities for roughly a century. Some of the earliest Play Streets resulted from a focus on reducing pedestrian-motor vehicle crashes, while also providing a play space for people in crowded urban areas [21, 29]. Most of the Play Streets during this time were coordinated by city agencies [19–21, 29], and were highly structured, with specific instructions for set-up [29] and games offered (e.g., group sports, individual sidewalk activities) [21]. Success of these Play Streets was measured by counting the number of children who registered for Play Streets and the number who attended [21]. Parents were not usually present since activities catered to children and staff were on hand to supervise [21, 29].

The focus of Play Streets began to shift in the 1970s to creating a social neighborhood environment for children and adolescents, and coordination was assumed by block and/or neighborhood associations [30, 31]. Surveys of Play Streets participants were the main way they were evaluated [19, 20], and results indicated that Play Streets increased social engagement and reduced the immediate need for a permanent playground in the community [31]. After these efforts, Play Streets began to spread to large, densely populated cities, with local organizations and government agencies often leading implementation [31].

Although Play Streets have been implemented across multiple decades, their recent resurgence highlights a need to better understand their impacts and the evidence base for their implementation. To address this gap, the primary aim of this study was to document how Play Streets impact opportunities for safe active play and physical activity for children and adolescents. Secondary aims were to describe neighborhood and community impacts and examine evaluation methods used in each study in order to inform future evaluation research as Play Streets continue to grow in popularity.

## Methods

### Search strategy

A systematic literature search was conducted using the following databases: PubMed, PsycINFO, Web of Science, Academic Search Complete, Cumulative Index to Nursing and Allied Health Literature (CINHAL), and Google Scholar. Given the possibility of overlap with similar initiatives (e.g., pop-up parks, open streets, ciclovias), search terms were broader than “play streets”. Each search used the following terms: “play street*” OR “pop-up park” OR “open street*” OR “ciclovia*” OR “mobile physical activity*.” The search aimed to identify peer-reviewed articles published worldwide, in English, through December 2017. Relevant references cited in each study were also reviewed for inclusion. Preferred Reporting Items for Systematic Reviews and Meta-Analysis (PRISMA) guidelines were used for tracking articles identified through the literature search [32] to ensure a systematic approach to documenting the search process.

### Study selection

Two researchers conducted independent searches of the aforementioned databases, reviewed titles and abstracts for potential papers, and reviewed complete texts to determine the final sample. Of the potential papers, intervention studies were included that had one of the following: (1) an explicitly stated Play Streets intervention, (2) a Play Streets-style intervention with temporary closure of a street or parking lot, or (3) an Open Streets/Ciclovía intervention with description of a specific physically active child’s play area as an activity hub, all of which did not charge for admission, were open to the general public, and did not allow traffic on the street or area. A Play Streets-style intervention was defined as the closing down of a street or parking lot to traffic to provide the public with a safe, open space to actively play and/or be physically active that was accessible at no cost, was designed primarily for youth (children and/or adolescents), and may have organized events and environmental supports such as marked play areas, loose equipment, and games. Ciclovía or Open Streets interventions were only included if they contained a Play Streets-style component in addition to the broader streets event, and reported specific results describing this sub-component of the event.

Intervention studies were excluded if they described Play Streets interventions but did not include process, impact, or outcome evaluation data about the Play Streets intervention; did not examine impacts on children or adolescents; or if a Play Streets-style intervention or sub-component of a broader event did not implement or measure child or adolescent focused active play activities (e.g., if the description of Ciclovía or Open Street activity hub did not include this information or detail, the article was excluded). Since Play Streets involve temporary changes to support active play, interventions exploring permanent changes to the built environment were also excluded. Discrepancies regarding article inclusion (*n* = 12) were resolved through consensus between the two researchers (author 2 and 3).

### Data extraction and validity checks

An Excel spreadsheet was created to extract information about the intervention, sample, methods, study design, measures, and limitations. Measures included impacts on opportunities for safe active play and physical activity, and neighborhood and community impacts. One researcher (author 2) extracted data for each article. Two researchers (author 1 and 5) extracted data from one article to ensure consistency in the extraction process across researchers. A second researcher (author 4) conducted a quality check of a randomly selected subset (15%) of articles included for data extraction; this quality check was confirmed by a third researcher (author 5). There were few discrepancies in data extraction (only in the level of detail provided), and these were resolved by consensus. Extracted data are presented in summary tables, and the findings qualitatively described.

Study quality was examined for all studies using a modified version of the Cochrane Collaboration’s assessment tool. The Cochrane tool assesses risk of bias in randomized control trials (RCTs) across 7 categories: sequence generation, allocation concealment, blinding of participants and personnel, blinding of outcome assessment, incomplete outcome data, selective outcome reporting, and other sources of bias [33, 34]. Since the Cochrane tool was developed for assessing risk of bias in RCTs, we adapted the tool and removed the first four categories, since they are not relevant for non-RCTs. Risk of bias assessments were conducted by one author, with confirmation of assessments conducted by a second author.

## Results

**Fig. 1:**
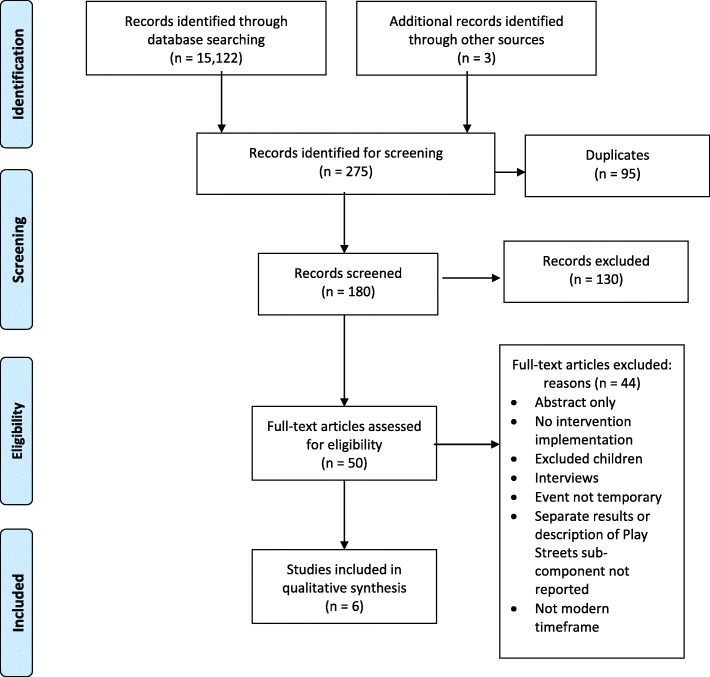
PRISMA diagram of literature search and selection through December 2017. *Note.* Based on a systematic literature review conducted on peer-reviewed intervention studies published worldwide, in English, through December 2017 that documented free-to-access Play Streets or other temporary spaces that incorporated a designated play area (Play Streets-style interventions)

The initial literature search identified 15,122 articles, with three additional articles identified through researcher recommendation or in review of article reference lists. Of these 15,125 initial articles, 275 were identified as relevant through reviewing article titles. Duplicates were removed (*n* = 95), leaving 180 articles for abstract review. After abstract review, 50 articles were included for full text review. Six articles met inclusion criteria and were included in the final synthesis. The PRISMA flow diagram in Fig. 1 illustrates the article selection process. Three of the articles excluded during the full text review focused on early Play Streets efforts (1920s–1930s) and were therefore discussed in the background section of this paper to provide historic context. Results from current Play Streets efforts (*n* = 6) are presented as either a specific Play Streets intervention or a Play Streets-style intervention with temporary closure of a street or parking lot. Any articles detailing broader events, such as Ciclovías or Open Streets, that did not specifically mention incorporating an activity hub/area were excluded. Seven Ciclovía or Open Street events mentioned having activity hubs/areas (e.g., fitness classes, children’s activities, school games, etc.…) that could have included an area with active play opportunities for children or adolescents; however, none of these papers included enough detail to determine if this occurred, and were therefore also excluded from this review [24, 35–40]. One of these studies also mentioned creating temporary park spaces from parking spots; however, these were not well-described and therefore the article was not included [37]. Additional articles were excluded if an intervention was not implemented, if children or adolescents were excluded, if the event was not temporary, and if the study was not offered in a modern timeframe. Intervention types meeting inclusion criteria were Play Streets (*n* = 4), a pop-up park (*n* = 1), and a mobile physical activity unit intervention (n = 1). Four of the six included studies used cross-sectional designs, and two used a pre-post test design (quasi-experimental and non-equivalent). Table 1 summarizes each study’s location, population, and intervention. Table 2 presents a detailed description of the measures and outcomes from each study, including outcomes for active play, physical activity, and neighborhood and community impacts. The following sections qualitatively describe impacts on opportunities for play, physical activity, and environment, separately identifying these for Play Streets and for Play Streets-style interventions.

**Table 1:** Locations, populations, and interventions for Play Streets and Play Streets-style interventions

Reference	Location, Setting	Sample Description			Design	Intervention Description
		N	Age	Gender, Race/Ethnicity, SES, BMI		
Play Streets						
Cortinez-O’Ryan et al. (2017) [41]	Santiago, Chile Low-middle income neighborhoods with children ages 4–12.	Mean number of Attendees *n* = 60 (sd = 22, range: 29–126); Pedometers: n = 100 (51 intervention neighborhood). Intervention and comparison participants were statistically similar other than age.	Attendees: Age: 4–12 yrs Pedometers: 4–8 yrs Intervention children: 41% 4–8 years, 58.8% were 9–12 years. Comparison participants were significantly younger than intervention participants (65% were 4–8 years of age).	51% girls, 100% Latin, 75% classified as low socio-economic position; 55.5% overweight or obese.	Quasi-Experimental: pre-posttest with comparison neighborhood	Street Play Initiative: “Juega en tu Barrio” (Play in your Neighborhood): closing 4 consecutive blocks for children to increase physical activity and outside play. Held 2/week (Wed. & Fri.) for 12 weeks from Sept.-Dec. 2014 from 17:30 to 20:30 with adult supervision (*n* = 26 total). All families with a child received a self-monitoring/reminder calendar and play materials (ropes, kites, paddleballs, diabolos (juggling), and balls). Local adult monitors led group games and incentivized children to meet each other during 1st 4 sessions. Stewards from CicloRecreo Via rerouted traffic with uniforms and identifiable signs.
Murray & Devecchi (2016) [43]	Winterborough of Hantown, England, UK 5% most socio-economically deprived areas in England	*n* = 216 surveys (response rate = 216/1000); n = 25 semi-structured interviews.	Surveys: *n* = 148 local adults, *n* = 68 children; Interviews: *n* = 7 parents, *n* = 11 children at event, *n* = 7 children via phone.	81% lived within 1 mile of Street Play project, 56% residents of the borough	Cross-sectional: surveys with adult and child sections (3 languages: English, Polish, Arabic); Semi-structured interviews	Street Play Project: “Hantown Street Play Project”: 1 pedestrian street: 16 consecutive Tuesdays June-Oct. 2013 3:30–5:30 pm. Traditional games were set up and supervised. Street was already closed to traffic prior to project.
Zieff et al. (2016) [16]	San Francisco, CA	SOPARC: *n* = 1116 Comparison non-Play Street: *n* = 248 Surveys: *n* = 75	SOPARC: 54.5% adults, 38.4% children (≤14 yrs. out on streets) Comparison: 87.7% adults, 4.9% children (≤14 yrs. out on streets) Surveys: 100% adults	SOPARC: 30.3% Latino, 28.1% Black, 23.5% white. Comparison: 57.2% Black Surveys: 8.0% Asian, 25.3% Black, 14.7% Hispanic, 34.7% white, 5.4% < high school graduate	Cross-sectional: SOPARC observations; Adult surveys	Play Streets: 1–2 city blocks closed to motorized traffic on weekends for 4 h to create an open place to play and do leisure physical activity summer of 2013. 1 of 8 sites funded by Partnership for a Healthier America.
D’Haese et al. (2015) [17]	Ghent, Belgium	Accelerometers: *n* = 126 (intervention Play Streets street *n* = 54, control non-Play Streets street *n* = 72)	Intervention: mean age = 8.7 ± 2.2 yrs.	Intervention: 59.3% boys; 38.9% low family SES; 81% lived in Play Streets boundaries, 19% lived nearby the Play Streets area.	Non-equivalent pre-posttest design (both groups): accelerometers (8 days: 4 days non-Play Streets week, 4 days Play Streets week or vice versa); parent pre-post questionnaire	Play Streets: Prohibit car traffic and have street(s) open for children’s play, mainly to encourage free play. Play Streets (*n* = 19) included in study were held for at least 7 consecutive days from 2 to 7 pm in July and/or Aug. 2013 (Play Streets could happen a max of 14 total days in July and/or Aug., consecutive or not). 3 volunteers mandatory/Play Street, could “hire” for free a box of play equipment from city council, other play materials, hire an organized activity by city council, or organize activities themselves.
Play Streets-style intervention with temporary closure of a street or parking lot						
McGlone (2016) [22]	Melbourne, Australia (Albert Park: affluent suburb of Melbourne) Pop-up Park users	Semi-structured Child interviews *n* = 20) Focus groups: children *n* = 9, adults n = 7	Child interviews: 5–12 yrs.	Child interviews: 75% female (*n* = 15) Focus groups: child 77.8% female (n = 7), adults 100% female (n = 7); local residents and staff of Albert Park Primary School	Cross-sectional: teacher semi-structured interviews; 2 focus groups (adult and child)	Pop-up Park: 12–24 month trial (beginning July 2013) of a pop-up Park near a primary school, open at all times to the general public.
Espinoza et al. (2012) [42]	Santa Ana, CA Specific neighborhood (92,701 zip code of Santa Ana, CA) that lacked access to indoor recreation, exercise facility, or outdoor play area (> 70% lived ≥20 min from one of these locations).	N = 24 families with children ages 6–14 yrs	Children: 53% were 6–10 yrs	Children: 53% male; 84% Latino/Hispanic; 92% annual income < $30,000; 88% lived in an apartment	Cross-sectional: non-random area sampling	Mobile Physical Activity Unit (MPAU): Abandoned bus was renovated and filled with playground equipment to create a MPAU, which was intended to “bring the playground” to participating families and allow children an opportunity to play in a safe and supervised environment. MPAU driven to a single school every Tuesday evening from 4 pm–6 pm and Saturday mornings from 10 am-12 pm for a total of 12 weeks. Children were assigned to one of two groups for play: 6–10 yrs. old and 11–14 yrs. old (given colored jerseys corresponding to each age group). Children could participate in a total of 3 different games and/or activities (selected and supervised by the research team and volunteers) along with 30 min of free play. Drinks and orange slices were provided.

*NR* not reported, *yr(s)* year(s), *hr(s)* hour(s), *n* sample size, *SD* standard deviation, *approx*. approximately, ‘~’ approximately, *CA* California, *SES* socioeconomic status, *UK* United Kingdom, *US* United States

*Note.* Based on a systematic literature review conducted on peer-reviewed intervention studies published worldwide, in English, through December 2017 that documented free-to-access Play Streets or other temporary spaces that incorporated a designated play area (Play Streets-style interventions)

**Table 2:** Methods and outcomes for Play Streets and Play Streets-style interventions

		Outcomes for Key Domains		
Reference	Methods	Active Play	Physical Activity	Neighborhood and Community
Play Streets				
Cortinez-O’Ryan et al. (2017) [41]	Wrist pedometers (children) 1 week accept water activities (baseline, final: 12th–14th week), parental surveys (baseline, final: 12th–14th week, 86% mothers), systematic counts of play every hour of intervention, 8 semi-structured interviews (3 pre, 5 during, 8 post), 4 focus groups (2 pre, 2 post).	Mean attendance n = 60 (SD = 22, reach =34% of neighborhood kids, 58% of participants were girls). Peak attendance was reached towards latter part. 24 (92%) of Play Streets were implemented as planned. Most commonly used play materials: balls and jump ropes (primarily used in activities guided by adults-96%). Interviews with adults: children only play on block where they live (parental permission/trust of own block), neighbors wanted street play intervention to continue longer, but it was not. Parent survey: significant increase in number of weekdays with outdoor play for intervention participants, after-school outdoor playtime, and weekly outdoor playtime after-school. Overall intervention cost = USD $2275. Parent surveys: primary motivation for outdoor play = presence of other children (59%), street play replaced screen time for 62% of children.	Pedometer: significantly more steps from baseline to final assessment in intervention participants (Monday to Sunday) and during the 3-h intervention. Significant increase in intervention children meeting pedometer-derived physical activity recommendations from baseline to final assessment. No significant differences for steps on intervention days were found. Control participants had no significant differences from baseline to final assessments for steps.	Comments during session: *n* = 16 supportive comments from neighbors, *n* = 5 complaints (mostly noise), *n* = 26 car drivers complained about traffic detours. Traffic stewards increased perceived safety, viewed as “eyes in the street”. Parent surveys: baseline main reason parents did not allow street play for child was traffic/stranger danger (76%); baseline 4% of children had permission to play in street without supervision, 65% had permission when street was closed to traffic; baseline 35% agreed that neighborhood was safe for children to play during daytime, 54% agreed during final session. 30% of intervention parents reported meeting new neighbors, 54% strengthened relationships with neighbors previously met. How was it useful for children: 36% child was more sociable/more friends; 28% child more independent/confident.
Murray & Devecchi (2016) [43]	Resident surveys (child and adult). Semi-structured interviews with residents (parents and children) during an event or via telephone. Field notes.	Field notes: mean attendance *n* = 14.66 (SD = 6.2, range: 8–33), 50% boys; 1 rainout with *n* = 0 attendance. Play was planned, resourced, initiated, led and supervised by project adults, project adults played with children during sessions to ensure play. Children and parents identified activities. Interviews: 56% had not attended (timing conflicted or did not know about it). All interviewees with child attendee said child liked it. “enjoyment” was liked most (29%). Preference for activity linked to mastery (36% of children). 43% of parents said without the street play project that their children would be indoors; 86% said children do play outside even without project. 71% of parents / 43% residents valued project because it provided safe and supervised outdoor play for local children. Surveys: 68% were not aware of the project, 32% who were aware found out through word of mouth, school fliers, street notices, project workers. 61% thought street play project was a good opportunity for children to play safely outdoors. Parent surveys: Activities played by children: parachute, coloring, skipping, snakes and ladders, counting on the rockets, hula hoop, races, marbles, dice (often used as a football), ball, cycling (own bikes), “stuck with sellotape”, getting exercise, “lots of things”. Child surveys: freedom/new activities preferred (80% said they liked new or different play equipment; 24% liked learning new games/activities)	NR	Surveys: Social interaction opportunities provided by project were valued by parents, children, and residents; most residents said project helped children and adults interact more. Street play was identified as: 61% a good way for children to make new friends; 56% a good way for children to feel part of the community, 28% a good way for neighbors to get to know each other better, 20% it led to a better sense of community. Interviews: 43% of parents identified social interaction as the main reason they liked the project.
Zieff et al. (2016) [16]	1) Adult surveys, 2) System for Observing Play and Recreation in Communities (SOPARC), 3) Google Earth Pro and maps from City of San Francisco website (1/4 mile radius around each Play Streets location)	Attendance NR. Adult survey respondents most liked: free place to exercise (34%); convenient location (32%); and a place for social interaction (24%). 36% attended to be physically active, 50% reported climbing wall as favorite activity, 97% said they would attend again. % impact of added open space in relation to existing open space: added space was primarily a result of streets closed to motorized vehicles, but also included 1 closed parking lot. There was an increase of open space ranging from 47 to 100% (47% in Tenderloin, 50% in Bay view, and 100% in Excelsior). Activities included climbing wall, bicycle ramps, and spontaneous activities (magic show, basketball, soccer, tag, bean bag throw, sidewalk chalk drawing, Zumba, and hula hoops).	During Play Streets, the majority of children ≤14 yrs. of age engaged in some non-sedentary activity; children were engaged in vigorous activity more than other age groups; accompanying adults were engaged primarily in sedentary behavior; many female teens were sedentary. Play Streets increased the proportion of people who were engaged in vigorous physical activity by 23.1%, but also increased proportion of people engaged in primarily sedentary behavior by 24.7% (mostly accompanying parents who sat and watched children). During non-Play Streets, fewer people were seen and most activity was walking (65%).	Adult surveys: 94% agreed or strongly agreed that Play Streets strengthens their community.
D’Haese et al. (2015) [17]	1) Child’s accelerometer data (8 consecutive days of wear: 4 non-Play Streets days, 4 Play Streets days) for both Play Streets and non-Play Streets children, 2) Pre-post parent surveys	Attendance NR. Parent Surveys: Of parents whose child played at Play Streets 62.5% reported daily use of Play Streets, 6.3% used the Play Street every weekday, 15.6% used it 1/week. 75.0% totally agreed that their child was enthusiastic about the Play Street, 59.4% perceived their child played more outside during the Play Street as usual.	Accelerometers: Significant differences in sedentary time and moderate-to-vigorous physical activity were found between a normal week and an intervention week. In intervention streets, sedentary time was less (137.7 mins/day vs. 146.3 mins/day) and moderate-to-vigorous physical activity was higher during the intervention condition (35.8 mins/day vs. 26.7 mins/day). In control streets, sedentary time was higher (164.6 mins/day vs. 156.5 mins/day) and moderate-to-vigorous PA was lower (24.3 mins/day vs. 26.9 mins/day).	Parent Surveys: 78.2% rather to totally agree that their child had a lot of friends in the Play Street; 71.9% rather to totally agree that it was safe to play in the Play Street for their child; 59.4% felt they had more social contact with neighbors thanks to the Play Streets;
Play Streets-style intervention with temporary closure of a street or parking lot				
McGlone (2016) [22]	1) Teacher supervised semi-structured interviews with children that used pop-up park, 2) Children’s focus group, 3) Adult focus group, 4) Observations	Attendance NR. All participants viewed Pop-up Park as “fairly important” or “very important” to the community; most children liked that there was a flexible space with no traditional play equipment; > 1/2 the children enjoyed the freedom of the set up; children used space for relaxation, semi-structured play, a place to enjoy nature. Primary themes from study: full barrier fencing is needed for safety, signage needed to be improved, recommended softer ground to reduce injuries, adult supervision is important for safety, seating is needed for adults, children preferred for space to remain flexible without any traditional park equipment (e.g., slides), space provided a different vantage point of community, some local residents expressed that it was a nuisance. Adult opinion: temporary space provided respite for some children and fostered creativity given lack of structure.	NR	Child Focus Group: Increased connection to the community was expressed; few expressed negative response by residents, although some conflict was experienced; pop-up park provided a different view of public life than other places. Child & Adult focus groups: all viewed space as fairly to very important to the local community due to need for more gathering space or children’s enjoyment of having contact with other people in community.
Espinoza et al. (2012) [42]	Baseline data collected via questionnaire administered in the home (and in Spanish), along with informal feedback collected from children and parents before, during, and after 12-week intervention period, to document barriers, aesthetics, proximity and availability of parks, open spaces or green belts in the 92,701 zip code, and the time it takes to walk to the nearest PA amenity. Daily attendance logs were collected to document children’s utilization of the MPAU.	Overall attendance was NR. During the 12 weeks, 100% of the children surveyed (n = 24) participated during weeks 1, 4, 10, and 12. 62% of the kids did not miss a session and during week 11, 25% (n = 6) children were absent. The study stated that comments from several parents during the informal interviews clearly demonstrates the need for this intervention in areas where there are very limited open spaces and/or parks. Children reportedly had “very positive and encouraging comments about their desire to play and be physical active”. Many children reportedly wanted the project to be held seven days a week instead of two, and some of the parents were also described as expressing this feeling.	NR	One parent reported that she “no longer worried about her child when they came to participate in the MPAU” (worry was from an incident where her child was hit by a car when playing in front of her home). Other parents felt that the MPAU provide a healthy and safe environment and that the volunteers served as great role models. Authors also reported an unexpected outcome: a parent approached one of the project staff and expressed her interest in developing exercise classes for the parents as well. Community involvement was cited as one of the contributing factors to the success of the MPAU.

*NR* not reported, *hrs* hours, *mins* minutes, *MPAU* mobile physical activity unit, *n* sample size, *PA* physical activity, *SD* standard deviation, *USD* United States dollars

*Note.* Based on a systematic literature review conducted on peer-reviewed intervention studies published worldwide, in English, through December 2017 that documented free-to-access Play Streets or other temporary spaces that incorporated a designated play area (Play Streets-style interventions)

As previously mentioned, all included studies were non-RCTs. In assessing the three Cochrane risk of bias features relevant for non-RCTs (incomplete outcome data, selective outcome reporting, and other potential sources of bias), two studies scored *low* risk across all three features [41, 42], two studies scored *low* in two features, but were *unclear* with regards to “incomplete outcome data” [17, 22], one study was assessed as *unclear* in all three features [43], and one study was assessed as *unclear* for “incomplete outcome data”, *high* risk of bias for “selective outcome reporting” due to a lack of data presented in the results, and *high* risk of bias for “other potential sources of bias” due to potential selection bias from differences in the two groups being compared [16] (see Table 3). Overall 55.5% of the risk of bias assessments were rated as *low* risk, 33.3% *unclear*, and 11.1% *high* risk.

**Table 3:**
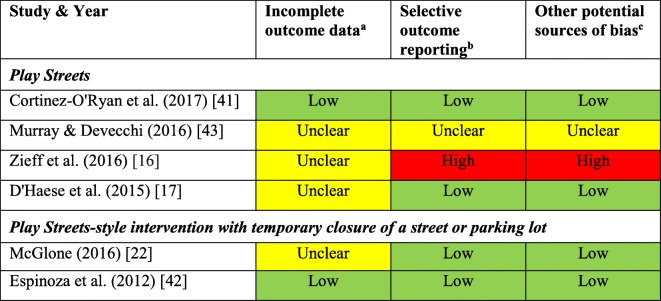
Summary of modified Cochrane risk of bias assessment for included studies

The first four Cochrane risk of bias assessment features are not relevant for non-RCTs, since all of the included studies were non-RCTs these were not assessed (random sequence generation, allocation concealment, blinding of participants and personnel, and blinding of outcome assessment)

^a^Attrition bias due to amount, nature or handling of incomplete outcome data

^b^Reporting bias due to selective outcome reporting

^c^Bias due to problems not covered elsewhere in the table

note: green cells indicate "low risk" of bias, yellow cells indicate "unclear risk" of bias, and red cells indicate "high risk" of bias

### Impacts on opportunities for active play

#### Play Streets

All of the Play Streets were described as creating safe places for children to play outdoors [16, 17, 41, 43]. This information was collected through a variety of methods across studies, including interviews, focus groups, and surveys with stakeholders, parents, and children, in addition to attendance and other field notes throughout the Play Streets. Attendance was only reported in two studies and ranged from μ = 14.66(SD = 6.2) in Hantown, England [43] to μ = 60(SD = 22) in Santiago, Chile [41]; the other studies only reported the number of study participants. Two main safety elements were described: areas were closed to motorized traffic thereby reducing traffic safety concerns and guardians or volunteers were present to provide adult supervision. Play Streets occurred once or twice per week, between 4 and 16 weeks, and mostly during summer months. One Play Street in Belgium occurred on 7 consecutive days [17]. Play Streets typically occurred on a single block or across several blocks. One notable exception in terms of location was a Play Street that occurred at a closed parking lot (the other Play Streets occurred on city blocks) [16].

Studies varied in terms of mentioning the types of equipment and activities that were used for play at Play Streets. When studies described the types of equipment and/or activities at the Play Streets, loose equipment and activities included balls, jump ropes, hula hoops, and opportunities for free play [41, 43]. In one study conducted in the U.K. 100% of parents whose children attended Play Streets said their child enjoyed it and 100% of children who attended Play Streets said they liked it [43]. Eighty percent of children liked being exposed to new play equipment and 24% to new games/activities [43]. In Ghent, Belgium, 75% of parents stated that their child was enthusiastic about Play Streets and 59.4% reported that their child played more outside during the Play Street [17]. Another study reported that children had very positive reactions and encouraging comments about their desire to play [43].

The impacts of Play Streets on opportunities for play were often ascertained via parent surveys, attendance logs, or interviews. Parents reported that children utilized these play spaces when available. In one study of Play Streets offered on at least 7 consecutive days, parents whose child played at Play Streets reported that nearly 63% visited it daily; 16% reported visiting Play Streets once per week and 6% reported doing so every weekday [17]. Another study reported that based on attendance logs, reach of the Play Street intervention was 34% of the children in the target neighborhood [41].

Overall, Play Streets created safe places for outdoor play. One study documented an increase in available open space ranging from 47 to 100% as a result of the Play Streets [16]. Parents who participated in a survey (*n* = 100) in another study noted that Play Streets led to a significant increase in the number of weekdays with outdoor play from 2 to 3 days (median, *p* = 0.001), after-school outdoor time play time from 60 to 90 min/day (median, 120 to 300 min/week (median, *p* = 0.02), and weekly outdoor playtime after-school (*p* = 0.01), with no changes for comparison [41], and pedometer data also supporting these changes. Pedometer data revealed that Play Streets also led to significant increases in the percentage of children in the Play Streets neighborhood meeting pedometer derived physical activity guidelines (12,000 daily steps for girls, 13,000 daily steps for boys) from 27.5 to 52.9% by the end of the 12 weeks (*p* < 0.01), with no significant changes for the comparison neighborhood. Adults who were surveyed in one study noted how the Play Streets provided an opportunity for a free place to exercise in a convenient location (i.e., on the block where the child lives) [41]. In addition, 43% of parents who participated in interviews (*n* = 25) noted that without the Play Streets, children would be indoors [43]. Sixty-two percent of parents from another study noted that the time spent at the Play Street replaced what would have been usual screen time for their children [41]. In another study, 71% of parents felt that Play Streets provided safe and supervised outdoor play for local children and 61% thought the Play Streets project was a good opportunity for children to play safely outdoors [43].

#### Play Streets-style interventions with temporary closure of streets or parking lots

Two studies were identified that described Play Streets-style interventions with temporary closure of a street or parking lot. Data describing active play opportunities in these interventions were collected through a variety of methods in both studies, including interviews, focus groups, and surveys with stakeholders, parents, and children, in addition to attendance and other field notes. In one study, in Melbourne, a play area was established as part of a pop-up park [22]. A pop-up park, with partial fencing, was installed on the road in front of a primary school for a 12–24 month trial; it was open at all times to the general public. Observations and qualitative data indicated that children were active through dance, jumping ropes, and doing flips and cartwheels. The investigators concluded that the pop-up park successfully created opportunities for unstructured play.

The second study explored the implementation of a Mobile Physical Activity Unit (MPAU) in a predominantly Latino/Hispanic neighborhood in California [42]. A MPAU is a renovated bus with playground equipment, which was intended to give children a “playground” that was non-existent in their neighborhood. The MPAU was driven to a single school every Tuesday evening from 4 pm–6 pm and Saturday mornings from 10 am-12 pm and situated in a parking lot for a total of 12 weeks. Because of the lack of parks and playgrounds, this intervention created a temporary, yet recurring, place for children to play. Attendance records showed that children in the study regularly visited with MPAU. According to brief informal interviews conducted throughout the 12-week program with parents and children, children had very positive and encouraging comments about how the MPAU created opportunities for play and fun. Children also reportedly wanted the MPAU to be available more often than it was during the 12-week period.

### Impacts on physical activity

#### Play Streets

Of the four studies specifically describing Play Streets [16, 17, 41, 43]; physical activity outcomes were only reported in three of these; one used the System for Observing Play and Recreation in Communities (SOPARC) systematic observation and adult surveys [16], and two used device-based and self-report measures, either pedometers, surveys, interviews, and focus groups [41] or accelerometers and pre-post parent surveys [17].

The most recent study was conducted in 2014 in Santiago, Chile [41] and measured physical activity using pedometers in a pre-posttest quasi-experimental study. Play Streets were held 2x/week for 12 consecutive weeks; participants had significantly more steps from baseline to final assessment and during the 3-h Play Streets intervention time, with non-Play Streets participants experiencing no differences. There was also a significant increase in the number of Play Streets children meeting pedometer-derived physical activity recommendations from baseline to posttest.

The other two studies reporting physical activity outcomes were conducted in 2013, with one in the U.S. conducted in four different neighborhoods of San Francisco, California [16]. Physical activity was examined using SOPARC to conduct observations and measure participants’ type of physical activity. The authors concluded that more active play and physical activity occurred on streets when Play Streets were offered as compared to days when Play Streets were not offered, with an average of 11.3% more children participating in vigorous physical activity across all Play Streets communities as compared to non-Play Streets comparison streets [16]. This study revealed that 38.4% (*n* = 429) of Play Streets attendees were children and 7.1% (*n* = 79) were adolescents. SOPARC demonstrated that most children who attended Play Streets (≤14 years of age) engaged in some non-sedentary activity and that children were engaged in vigorous physical activity more than other age group attendees. An average of 1.9% more children participated in moderate physical activity across all Play Streets communities, as compared to non-Play Streets comparison streets [16]. Activity for adolescents was less clear, with 0.9% of Play Streets adolescents engaging in more vigorous physical activity than non-Play Streets teens and an average 2.5% fewer adolescents engaging in moderate physical activity at Play Streets. However, on average across all communities, 12.1% more adults on a street during a Play Street engaged in sedentary behavior than adults on a street on a non-Play Streets day. This was described as being due to the majority of adults being parents accompanying children on a Play Streets day as compared to the majority of adults walking for transportation or purposes on a non-Play Streets day.

The third Play Streets intervention reporting physical activity outcomes, conducted in Belgium [17], used accelerometers to measure children’s physical activity movement during 4 days with Play Streets and compared these to physical activity levels on 4 days without Play Streets, and a comparison sample completing the same measurement protocol (without a Play Streets intervention). About half of participants were boys, one-third were from low socio-economic status families, and the mean age was 9 years. The study showed that Play Streets increased overall moderate-to-vigorous physical activity by 9.1 min/day and reduced sedentary behavior by 8.6 min/day in children between a normal week and a Play Streets week, as compared to youth in the control group where sedentary time was greater and moderate-to-vigorous physical activity time was lower.

The final Play Streets intervention was conducted in England, U.K. [43] and physical activity outcomes were not reported. Physical activity outcomes were also not reported for Play Streets-style interventions with temporary closure of a streets or parking lots or for Play Streets subcomponents of broader events with a Play Streets-style subcomponent.

### Neighborhood and community impacts

#### Play Streets

Neighborhood and community impacts were reported via surveys, interviews, and/or focus groups in all four Play Streets studies. Play Streets strengthened current relationships with or meeting new neighbors [41, 43], increased social interactions and connections [17, 43], increased social interactions and friends for children [17, 41, 43], increased independence of children [41], and created a better sense of community [16, 43] or a strengthened community [16, 43]. Play Streets also increased open space in the community [16] and perceived safety [17, 41]. Five complaints were reported by neighbors regarding noise and 26 car drivers in the community complained about traffic detours for Play Streets [41].

#### Play Streets-style interventions with temporary closure of streets or parking lots

Neighborhood and community impacts in the two Play Streets-style interventions were also reported using surveys, interviews, focus groups, or through informal feedback. Play Streets-style interventions also increased social interactions and connections [22], while noting the provision of a different view of the community [22] and the provision of positive role models, increased community involvement, and increased interest in developing additional opportunities for community engagement [42]. The MPAU intervention increased perceived safety [42]. A complaint revealed through a focus group was that the pop-up park was perceived as a nuisance by some in the community [22].

## Discussion

Refereed literature documenting impacts of Play Streets and Play Streets-style interventions is limited. As shown in this review, most published studies describing the impacts of Play Streets or Play Streets-style interventions have been written from a general outcome evaluation perspective. Although these studies provide some evidence and support for Play Streets as a potentially effective approach to increase active play and physical activity in under resourced communities, there is a gap in knowledge regarding effective implementation and related impacts. Current evidence suggests that Play Streets are not only about increasing physical activity, but also about other important associations, having the potential to strengthen communities. This review identified six quasi-experimental studies assessing Play Streets, and although risk of bias was relatively low in over half of the risk assessments, physical activity and community outcomes were inconsistently and rarely measured across the studies, limiting conclusions to promising impacts and associations. The search process for this systematic literature review revealed that Play Streets are not new, with this systematic literature review revealing multiple purposes of current Play Streets. These include enhancing or improving safety; health; physical activity and active play opportunities for children, adolescents, and families; social cohesion; and community capacity. These current impacts are broader than the intended impacts of initial Play Streets, which was to reduce pedestrian fatalities from vehicles. Play Streets included in this review had notable social and emotional impacts on participants, including increased social interactions and positive feelings about participants’ communities. These findings suggest great potential for broader community impacts beyond the individual level. There is a lack of evidence regarding sustainability and the long-term benefits of Play Streets, economic costs, and more objectively measured environmental impacts, including air quality and community violence, as well as social cohesion. Future work should examine these areas and publish this information in the peer reviewed literature to help build this evidence base.

Emerging evidence from this systematic review suggest that Play Streets and Play Streets-style interventions have the potential to increase active play and physical activity opportunities for children and possibly adolescents, by providing usable space for recreation in close proximity to residents that would not be available or accessible otherwise. When measured, evidence demonstrates practically meaningful increases in physical activity levels during these events, even suggesting that children and adolescents may be more likely to meet physical activity guidelines by participating in Play Streets. However, despite this potential association, evidence is limited. Current evidence where physical activity is measured suggests that Play Streets might be more effective at increasing vigorous physical activity as compared to moderate levels of physical activity. Future work should further examine whether vigorous physical activity should be the focus of Play Streets, or if there are ways to incorporate additional activities to encourage both moderate and vigorous physical activity. While promising, more research is needed to determine if physical activity levels are higher on days when Play Streets occur versus days when they do not occur, if increases in physical activity levels during Play Streets are meaningful from a public health perspective, and if Play Streets are well suited for both children and adolescents. Future work should also consider working with adolescents in the community to identify activities that could be added to better engage adolescents in physical activity at Play Streets.

This review revealed that Play Streets and Play Streets-style interventions are popular and gaining momentum in urban communities, which raises questions about their implementation, utility, and feasibility in rural settings. People living in rural communities face unique obstacles to engaging in daily, routine physical activity, including dispersed land use, fewer walkable destinations, and scarcely available infrastructure, like parks and playgrounds where children and adolescents can engage in active play [44, 45]. As revealed by this review, there have been no published studies of Play Streets in rural settings, which is an area for future research. Moreover, very few studies described the race and ethnicity of the users of the Play Streets and Play Streets-style subcomponents, which also raises questions about implementing them in diverse communities. Based on the existing literature, it is unclear whether implementation teams actively sought input from diverse community members when designing the Play Streets. Future efforts should involve children, adolescents, and families in determining culturally appropriate activities when designing Play Streets.

Although standard and validated methods were utilized in many of the studies, specifically seen through systematic observations and validation checks, physical activity assessment often relied upon self-report measures or was absent. The heterogeneity of research methods used and/or information reported in the studies included in this review makes it challenging to fully understand the effects of Play Streets and Play Streets-style interventions. The bias risk assessment conducted and the types of study designs included provides some indication of the strength and quality of the research. Each of the included studies utilized analytic designs over more descriptive designs. Specifically, four of six included studies were cross-sectional designs, and two studies involved a pre-post test design (quasi experimental and nonequivalent), which were able to account for some important potential confounders. The variation in study design, measures, and reporting also make it challenging to compare results across studies. Future work needs to expand upon these methods to include objective, device-based, physical activity assessment in combination with the systematic observations and validation checks reported in current studies. Researchers and implementers should look to further examine effects on regular physical activity across Play Streets seasons using accelerometers to capture sedentary, moderate, and vigorous levels of physical activity. Play Streets researchers and evaluators should also report overall Play Streets attendance, sample size, and demographic information, specifically age, sex, and race/ethnicity, for Play Streets initiatives in addition to reporting this information for study participants. The heterogeneity of how this type of information was reported in current studies does not allow for a complete understanding of reach.

## Limitations

Although this systematic literature search was comprehensive and ranged across decades, inherent in any literature search is the possibility that relevant studies were missed. This is possible given inconsistencies in terms describing Play Streets and broader events with Play Streets-style subcomponents. Although we included a wide range of terms to minimize this possibility, we could have missed other studies using unique names/identifiers of events that would have met our inclusion criteria. Also, while it is possible that broader street events, like Open Streets and Ciclovías, could include a Play Streets-style component as part of an activity hub/area, current literature does not provide enough detail to determine if this is happening, even when an article mentions that activity hubs were included as part of an Open Street or Ciclovía event. In addition, possible Play Street components that are part of larger events such as Open Streets/Ciclovías could have some differences in processes and resources needed to implement them; the social, environmental, and behavioral outcomes may be somewhat different as well. Future work should attempt to understand if Open Street and/or Ciclovía events include Play Streets-style components as part of the broader event and how these operate within this context. In addition, we did not include events where streets or parking lots were not completely closed off to traffic [46, 47]. Although we were intentional in not including these studies, there could have been some useful information around interventions strategies that was missed due to this exclusion. It is also possible that there are enhanced types of joint-use agreement interventions, [48] and interventions that specifically recruited participants for open play space events, [42] that were not included in this review that could contain information useful to developing Play Streets interventions.

We also acknowledge the potential for publication bias, as manuscripts unavailable in English were excluded. Publication bias may have also resulted from our decision to exclude studies from the grey literature, which if included could have increased the sample of articles and might have provided useful information about implementation and sustainability given that Play Streets have been implemented in dozens of cities globally such as Kensington and Victoria in Melbourne, Australia; Edinburgh, Scotland; Hackney, U.K.; Chicago, Illinois and New York City, NY in the U.S.. However, these articles were excluded because this review was focused on documenting impacts of Play Streets, which based on our initial review of the articles found from our search strategy, we felt would be in the scientific literature.

## Conclusions

This review fills an important gap in our understanding of Play Streets and other temporary Play Streets-style interventions as accessible intervention strategies for increasing physical activity opportunities for children and adolescents. As evidenced in this review, there is limited refereed literature describing how Play Streets impact active play, physical activity, and neighborhood and community outcomes, and even less literature describing implementation of Play Streets and Play Streets-style interventions. Although there is strength in current methods being used within the general outcome evaluations of the events reviewed here, there is a need to better understand planning and implementation procedures, specifically for Play Streets.

To advance scholarship regarding the implementation and impacts of Play Streets, future studies could explore the grey literature and conduct in-depth interviews with current and previous Play Streets’ implementers and participants to better understand implementation procedures. Future research should also go beyond descriptive and self-report measures. In addition, the following concepts should be included to help document and assess community impact of Play Streets: safety, social cohesion, implementation costs, social engagement, and sustainability. The following should be considered in study design: implementation measures, use, controlling for potential confounding factors, and systematically observed and device-based measured physical activity during Play Streets and non-Play Streets weeks, as well as how an entire Play Streets season impacts child and adolescent physical activity. Future work needs to further examine planning and implementation procedures of Play Streets and examine how Play Streets could be adapted for implementation in non-metropolitan, small town, and rural settings, and for diverse communities.

Play Streets hold promise as an effective strategy to strengthen communities and increase active play and physical activity by providing a safe and accessible space within communities. Given this promising evidence of increased physical activity rates reported across several studies, future work is needed to confirm these findings in different types of communities and to understand the barriers and facilitators to successful implementation.

## Abbreviations

CINHAL: Cumulative Index to Nursing and Allied Health Literature
MPAU: Mobile Physical Activity Unit
PRISMA: Preferred Reporting Items for Systematic Reviews and Meta-Analysis
SOPARC: System for Observing Play and Recreation in Communities
U.K.: United Kingdom
U.S.: United States of America
WHO: World Health Organization

## References

[1] Obesity and Overweight Fact Sheet [http://www.who.int/news-room/fact-sheets/detail/obesity-and-overweight].

[2] Ogden CL, Carroll MD, Lawman HG, Fryar CD, Kruszon-Moran D, Kit BK, Flegal KM (2016). Trends in obesity prevalence among children and adolescents in the United States, 1988-1994 through 2013-2014. JAMA.

[3] Global Recommendations on Physical Activity for Health [http://www.who.int/dietphysicalactivity/publications/9789241599979/en/]. 26180873

[4] Physical Activity Guidelines Advisory Committee (2008). Physical Activity Guidelines Advisory Committee Report, 2008.

[5] National Physical Activity Plan Alliance. 2016 United States report card on physical activity for children and youth. Columbia: National Physical Activity Plan Alliance. p. 2016.

[6] Australian Bureau of Statistics (ABS) (2013). Australian health survey: physical activity, 2011–12.

[7] Wijtzes AI, Verloigne M, Mouton A, Cloes M, De Ridder KA, Cardon G, Seghers J (2016). Results from Belgium’s 2016 report card on physical activity for children and youth. J Phys Act Health.

[8] Moreno L, Cano M, Orellana Y, Kain J (2015). Compliance of physical activity guidelines by Chilean low-income children: difference between school and weekend days and nutritional status. Nutr Hosp.

[9] Centers for Disease Control and Prevention (CDC) and World Health Organization (WHO). https://www.cdc.gov/GSHS/. Accessed 9 Mar 2019.

[10] Scholes S. Health Survey for England 2015: Physical activity in children. Health and Social Care Information Centre; 2016.

[11] Kerr J (2007). Designing for active living among children. A research summary. Fall.

[12] Taylor WC, Lou D. Do all children have places to be active? Disparities in access to physical activity environments in racial and ethnic minority and lower-income communities. In: Active Living Research: Robert Wood Johnson Foundation; 2011. https://activelivingresearch.org/sites/activelivingresearch.org/files/Synthesis_Taylor-Lou_Disparities_Nov2011_0.pdf.

[13] Play Streets [http://groundplaysf.org/projects/play-streets/].

[14] Play Streets! [http://www.seattle.gov/transportation/playstreets.htm].

[15] PlayStreets Chicago [http://www.worldsportchicago.org/programs/playstreets/].

[16] Zieff SG, Chaudhuri A, Musselman E (2016). Creating neighborhood recreational space for youth and children in the urban environment: play(ing in the) streets in San Francisco. Child Youth Serv Rev.

[17] D’Haese S, Van Dyck D, De Bourdeaudhuij I, Deforche B, Cardon G (2015). Organizing “play streets” during school vacations can increase physical activity and decrease sedentary time in children. Int J Behav Nutr Phys Act.

[18] Kuhlberg JA, Hipp JA, Eyler AA, Chang G (2014). Open streets initiatives in the United States: closed to traffic, open to physical activity. J Phys Act Health.

[19] United Kingdom Parliament (1938). Street Playgrounds Bill.

[20] United Kingdom Parliament (1935). Play Streets.

[21] Reeves WR (1931). Report of committee on street play. J Educ Sociol.

[22] McGlone N (2016). Pop-up kids: exploring children's experience of temporary public space. Aust Planner.

[23] Hipp JA, Eyler AA, Zieff SG, Samuelson MA (2014). Taking physical activity to the streets: the popularity of ciclovía and open streets initiatives in the United States. Am J Health Promot.

[24] Sarmiento O, Torres A, Jacoby E, Pratt M, Schmid TL, Stierling G (2010). The Ciclovía-Recreativa: a mass-recreational program with public health potential. J Phys Act Health.

[25] Playing out story [http://playingout.net/about/playing-story/].

[26] World Sport Chicago (2017). Search result for “playstreet”. Chicago sport blog.

[27] Play Streets [https://youtu.be/uc5ODQxK4Xo]. Accessed 9 Mar 2019.

[28] Alcaldia Mayor de Bogota. Ciclovía Bogotana. In*.*: Instituto Distrital de Recreacion y Deporte. https://www.idrd.gov.co/ciclovia-bogotana. Accessed 9 Mar 2019.

[29] Mullaney R (1938). Play streets. J Health Phys Educ.

[30] Reiss ML, Shinder A (1975). Play streets. J Leis Res.

[31] Reiss ML, Shinder AE (1976). Urban play streets: creating and operating part-time traffic-free zones. Transp Res Rec.

[32] Moher D, Liberati A, Tetzlaff J, Altman DG (2009). The PG. preferred reporting items for systematic reviews and meta-analyses: the PRISMA statement. PLoS Med.

[33] Higgins JPT, Altman DG, Collaboration TC (2011). Assessing risk of bias in included studies. Cochrane handbook for systematic reviews of interventions.

[34] Higgins JPT, Altman DG, Gotzsche PC, Juni P, Moher D, Oxman AD, Savovic J, Schulz KF, Weeks L, Sterne JAC (2011). The Cochrane Collaboration's tool for assessing risk of bias in randomised trials. Br Med J.

[35] Hipp JA, Eyler AA, Kuhlberg JA (2013). Target population involvement in urban ciclovias: a preliminary evaluation of St. Louis open streets. J Urban Health.

[36] Torres A, Steward J, Strasser S, Lyn R, Serna R, Stauber C (2016). Atlanta streets alive: a movement building a culture of health in an urban environment. J Phys Act Health.

[37] Wilson JD, Tierney P, Kim M, Zieff S. Temporary parks? Sunday streets, serving the need for urban outdoor recreation. J Park Recreat Adm. 2012;30(4):38–52.

[38] Wolf SA, Grimshaw VE, Sacks R, Maguire T, Matera C, Lee KK (2015). The impact of a temporary recurrent street closure on physical activity in new York City. J Urban Health.

[39] Sarmiento OL, Díaz del Castillo A, Triana CA, Acevedo MJ, Gonzalez SA, Pratt M. Reclaiming the streets for people: insights from Ciclovías Recreativas in Latin America. Prev Med. 2016;103:S34–S40. 10.1016/j.ypmed.2016.07.028. 10.1016/j.ypmed.2016.07.028 27497659

[40] Diaz del Castillo A, Sarmiento OL, Reis RS, Brownson RC (2011). Translating evidence to policy: urban interventions and physical activity promotion in Bogotá, Colombia and Curitiba, Brazil. Transl Behav Med.

[41] Cortinez-O'Ryan A, Albagli A, Sadarangani KP, Aguilar-Farias N (2017). Reclaiming streets for outdoor play: a process and impact evaluation of “Juega en tu barrio” (play in your neighborhood), an intervention to increase physical activity and opportunities for play. PLoS One.

[42] Espinoza A, McMahan S, Naffzinger T, Wiersma L (2012). Creating playgrounds, where playgrounds do not exist: a community based approach. Californian J Health Promot.

[43] Murray J, Devecchi C (2016). The Hantown street play project. Int J Play.

[44] Hansen AY, Umstattd Meyer MR, Lenardson JD, Hartley D (2015). Built environments and active living in rural and remote areas: a review of the literature. Curr Obes Rep.

[45] Umstattd Meyer MR, Moore JB, Abildso C, Edwards MB, Gamble A, Baskin ML (2016). Rural active living: a call to action. J Public Health Manag Pract.

[46] Ben-Joseph E (1995). Changing the residential street scene: adapting the shared street (woonerf) concept to the suburban environment. J Am Plan Assoc.

[47] Gill T (2006). Home zones in the UK: history, policy and impact on children and youth. Child Youth Environ.

[48] Farley TA, Meriwether RA, Baker ET, Watkins LT, Johnson CC, Webber LS (2007). Safe play spaces to promote physical activity in inner-city children: results from a pilot study of an environmental intervention. Am J Public Health.

